# Associations between interleukin-10, -12, and − 18 and periodontal health and disease: a cross-sectional study

**DOI:** 10.1007/s00784-024-05843-8

**Published:** 2024-07-31

**Authors:** Elif Ilke Cebesoy, Müge Altaca, Necla Asli Kocak-Oztug, Ilknur Bingül, Emine Cifcibasi

**Affiliations:** 1 Faculty of Dentistry, Department of Periodontology, Istanbul University, Istanbul, 34116 Turkey; 2 Institute of Graduate Studies in Health Sciences, Department of Periodontology, Istanbul University, Istanbul, 34126 Turkey; 3 School of Dentistry, Faculty of Health and Behavioural Sciences, The University of Queensland, Brisbane, QLD 4006 Australia; 4 Department of Medical Biochemistry, Istanbul Faculty of Medicine, Istanbul University, Istanbul, Turkey

**Keywords:** Periodontal disease, Gingival crevicular fluid, Interleukin-10, Interleukin-12, Interleukin-18, Inflammation, Cytokines

## Abstract

**Objective:**

We assessed the levels of Interleukin-10 (IL-10), Interleukin-12 (IL-12), and Interleukin-18 (IL-18) in the gingival crevicular fluid (GCF) of subjects with advanced periodontitis (SIII-SIV) compared to healthy controls and evaluated their correlations with clinical measurements.

**Methods:**

This cross-sectional study involved subjects (*n* = 60) diagnosed with stage III grade B-C (*n* = 13) to stage IV grade C (*n* = 17) periodontitis, and periodontally healthy controls (*n* = 30). Clinical periodontal measurements involved full-mouth. The concentrations of IL-10, IL-12, and IL-18 were determined using enzyme-linked immunosorbent assay (ELISA).

**Results:**

There were no significant differences in IL-12 level and IL-18/IL-10 ratio between the healthy and periodontitis groups (*p* = 0.413, *p* = 0.636, respectively). The IL-10 and IL-18 levels were significantly higher in the periodontitis group than in controls (*p* < 0.001, *p* < 0.001, respectively). Significant associations were observed between the periodontitis and IL-10 and IL-18 levels (OR = 1.46, %95 CI 1.19–1.795; OR = 1.13, %95 CI 1.059–1.207, respectively) (*p* < 0.001, *p* < 0.001, respectively).

**Conclusions:**

There was a correlation between pocket depth and the presence of IL-18 and a strong association between periodontitis and a high level of IL-18. However, there were no direct correlations among the three biomarkers and IL-18/IL-10 ratio, indicating that their roles in periodontal health are complex and multidimensional.

**Clinical relevance:**

Understanding the cytokine dynamics in GCF provides valuable insights into their potential clinical implications for periodontal disease diagnosis, risk assessment, and tailored therapeutic interventions.

**Supplementary Information:**

The online version contains supplementary material available at 10.1007/s00784-024-05843-8.

## Introduction

Periodontal diseases are multifactorial and chronic inflammatory diseases characterised by the destruction of tooth-supporting tissues. Their development and progression involve a complex interplay between the immune system and the periodontal microbiome. The new classification for periodontitis has a multidimensional staging and grading structure [1]. This structure assesses progression using categories (stages I–IV) based on disease severity, complexity, and extent. Stages I and II are the early phases, and stages III and IV are the most severe phases [2]. To reflect the biological characteristics of periodontitis, such as the risk of rapid progression and modifying factors such as smoking and diabetes, the stages are divided into grades A–C [2, 3].

Although the diagnosis of periodontal disease is primarily based on clinical measurements and radiographic findings, these provide insight only into the historical background of periodontal destruction rather than offering information on current disease activity, possible future and progression [4–6]. In this regard, the diagnostic and prognostic significance of immunoinflammatory mediators in gingival crevicular fluid (GCF) is important [5]. Assessing these biomarkers can provide information on the mechanisms of periodontal disease and its progression. Investigation of their relationships with the staging and grading of periodontal disease can enhance treatment of the condition [7].

Interleukin-10 (IL-10) is a potent anti-inflammatory cytokine that downregulates the production of certain pro-inflammatory cytokines and induces T-cell anergy [8, 9]. It is produced mainly by T cells, including Th0/1/2 cells, and also by B cells, macrophages, and monocytes [10]. Gingival inflammation initiates when bacterial lipopolysaccharides induce the synthesis of pro-inflammatory cytokines [11]. IL-10 inhibits the production of pro-inflammatory cytokines, such as IL-1/-6/-8/-12/-18, Interferon-γ (IFN-γ), and TNF-α [12–16]. These cytokines act as extracellular signalling molecules that facilitate IFN-γ production. However, IL-10 inhibits the production of IFN-γ [15]. IL-10 has been detected in the inflamed and healthy human periodontium. In vivo and human studies have demonstrated that deficiency in IL-10 accelerates alveolar bone loss. Thus, IL-10 may be crucial for maintaining periodontal health and preventing bone loss [17, 18]. In addition, IL-10 has anti-inflammatory and immunosuppressive properties in periodontal disease [11, 19, 20].

The bioactive form of interleukin-12 (IL-12) (p70) is a heterodimeric cytokine comprising p35 and p40 subunits, and is produced by neutrophils, monocytes, and macrophages [21, 22]. In addition to inducing cell-mediated immunity, this cytokine is the main mediator of the initial innate immune response to intracellular pathogens [21]. Its most important action is inducing the production of IFN-γ by Natural Killer (NK) and T cells, thereby fostering Th1 responses. Furthermore, IFN-γ and IL-12 are connected by a positive feedback mechanism [21–23]. As well as triggering an immune reaction, IL-12 may also contribute to the maintenance of immune function [23]. The mechanism of the effect of IL-12 on the progression of periodontitis is unknown. The ability of IL-12 to elicit both protective and destructive effects has been noted [24].

IL-18 is a pro-inflammatory cytokine in the IL-1 family and is produced by keratinocytes, activated macrophages, Kupffer cells, and osteoblasts [21, 25]. IL-18 was first classified as an IFN-inducing cytokine synthesised by NK and T cells. IL-18 and IL-12 synergistically stimulate IFN-γ production and the development of Th1 cells. However, IL-18 is not dependent on IL-12 for its activity [21]. Nevertheless, either IL-12 or IL-15 is needed for IL-18–mediated induction of IFN-γ production. This cytokine is essential for the conversion of naïve T cells into Th2 cells and stimulates the production of Th2 cytokines from basophils, mast cells, NK cells, and T cells in the absence of IL-12 [26]. This indicates that IL-18 can induce Th1 and Th2 responses. Orozco et al. reported that IL-18 was the most prevalent cytokine at gingival and periodontal disease sites, and local cytokine production increased with inflammation in the GCF [21]. The interactions of IL-10, IL-12, and IL-18 are implicated in the progression of periodontal disease [27]. However, no study has examined the relationships of these three cytokines with the current periodontitis classification.

Primary aim of this study was to compare the local cytokine reaction associated with the clinical periodontal situation by analysing the IL-10, IL-12, IL-18 levels, and IL-18/IL-10 ratio in the GCF of subjects with stage III–IV, grade B-C periodontitis and healthy controls, and investigate any correlations between these biomarkers and clinical assessments. Secondary aim of this study was to evaluate the relationships of biomarkers with clinical parameters according to periodontitis stage.

## Materials and methods

### Study design and the population

The study protocol was approved by Ethics Committee of the Faculty of Dentistry (FD) of Istanbul University (IU) (approval no: 2022/679625, file number:2021/70), and the procedures were conducted in compliance with the Helsinki Declaration (v. Nov. 2013). This cross-sectional clinical study was registered at clinicaltrials.gov (NCT05307068). After clinical and radiographic evaluations, subjects were classified into the periodontitis and control groups according to the 2017 Classification of Periodontal Diseases and Conditions [1, 6, 28]. In total, 30 subjects with stage III–IV (SIII–SIV) and grade B–C (GB–GC) periodontitis and 30 gingivally healthy subjects (controls) were recruited between April 2022 and June 2023 at the Periodontology Department of the FD of IU (aged 18–65 years). The periodontitis group included 7 SIII GB, 6 SIII GC, and 17 SIV GC subjects.

Subjects with at least 20 teeth, no known systemic disease, non-smokers, not taking antibiotics and/or using immunosuppressant drugs in the last 6 months, not taking medication, not having had recent periodontal therapy, not pregnant or breastfeeding, and having been diagnosed as having SIII-SIV GB-GC periodontal disease were assigned to the periodontitis group [6]. The subjects were informed of the study’s purpose and methodology, and their written consent was obtained prior to participation.

### Clinical periodontal assessments and GCF samples

Periodontal indices were recorded 1 week before collection of GCF. Clinical measurements included Plaque Index (PI) [29], Gingival Index (GI) [30], Bleeding on Probing (BOP [%]) [31], Probing Depth (PD), Clinical Attachment Loss (CAL), and Mobility index (MOB) [32]. A single calibrated researcher (EIC) conducted the measurements and documented the data using a Williams periodontal probe (Hu-Friedy, Chicago, IL, USA) at six locations on each tooth: mesio-buccal, mid-buccal, disto-buccal, mesio-lingual/palatinal, mid-lingual/palatinal, and disto-lingual/palatinal. For clinical measurements of the areas where GCF samples were taken, *L*, for *localised*, was added to the parameters (i.e., LPI, LGI, LBOP [%], LPD, LCAL, and LMOB).

GCF samples were obtained from the six deepest active periodontal pockets of subjects with periodontitis, whereas GCF samples from clinically gingivally healthy subjects were collected from six separate non-inflamed sites. Briefly, cotton pads were used to isolate the area, and cotton pellets were used to remove plaque. Subsequently, gentle air drying was applied from 20 cm. GCF samples were obtained using absorbent paper strips (Periopaper^®^, Proflow Inc., Amityville, NY, USA). After gently inserting the strips into the gingival sulcus/periodontal pocket until slight resistance was detected, they were left in position for 30 s. Blood-contaminated strips were discarded. Six strips per subject were collected, pooled, and stored at − 80 °C (Ultra Low-Temperature Freezer, New Brunswick Scientific) until required for laboratory testing.

### Measurement of IL-10, IL-12, and IL-18 levels

Enzyme-linked immunosorbent assays (ELISAs) were conducted to measure the IL-10, IL-12, and IL-18 concentrations in GCF samples at the Department of Medical Biochemistry of the Faculty of Medicine of IU. Eppendorf tubes each with 6 periopapers were allowed to equilibrate to room temperature for 20 min after thawing. Next, a volume of 450 µl 1% BSA-PBS-Tween buffer solution (Bovine serum albumin-phosphate buffered saline; pH: 7.4) was pipetted into the eppendorf tubes and incubated on a swinging platform overnight at 4 °C. Standards and samples of healthy and periodontitis subjects were pipetted into microplate wells, and analysis was performed in accordance with the manufacturer’s protocols (MyBioSource, San Diego, CA, USA). Absorbances of standards and samples were read at 450 nm by using a spectrophotometer (Biotech, UK). Standard concentrations and absorbances were used to calculate the IL-10, IL-12, and IL-18 concentrations (pg/mL) for samples taken from both healthy and periodontitis subjects.

### Statistical analyses

The sample size was determined using G*Power v. 3.1.9.6, based on the GCF IL-18 values from a reference article [33]. The 95% confidence level (1-α), 85% test power (1-β), and d = 0.8 effect size were considered, together with the two-way hypothesis and 30 subjects per group.

IBM SPSS v. 23 and AMOS v. 24 were used to analyse the data. The Shapiro-Wilk test was used to evaluate conformity to the normal distribution. The independent two-sample *t*-test for normally distributed data, and Mann–Whitney U-test for non-normally distributed data were used for comparisons. The Kruskal–Wallis and Dunn test were used to compare non-normally distributed data for groups of three or more, and one-way ANOVA and the Duncan were used for normally distributed data. The Pearson’s chi-square test and Yates’ correction were used to compare categorical data by group. A correlation analysis was conducted using Pearson’s and Spearman’s rho coefficients. Binary logistic regression was used to determine associations between clinical parameters and biomarkers. The logistic regression was performed as a Univariate model, and the effect of each independent variable was analysed separately from the other variables. The Hosmer-Lemeshow test was used to assess the reliability of the regression model. Results are shown as means ± standard deviations and medians (minimum–maximum) for quantitative data and frequencies (percentages) for categorical data. A value of *p* < 0.05 was taken to indicate statistical significance.

## Results

### Study population

The demographic parameters of the study population by age, sex, and number of teeth are listed in Table 1. The study included 60 subjects, 13 with SIII periodontitis (7 GB and 6 GC), 17 with SIV periodontitis (17 GC), and 30 healthy controls. The healthy group had a greater number of teeth than the periodontitis group (*p* < 0.009), and the periodontitis group was older than the healthy control group (*p* < 0.001). The sex distributions of the groups were similar (*p* < 0.789). Controls had more teeth than the SIV subgroup (*p* = 0.018) but not the SIII subgroup, and were younger than both of those subgroups (*p* < 0.001).

**Table 1:** Comparison of age and the number of teeth among the healthy group and the periodontitis group, and periodontitis subgroups

	**Healthy (H)**				**Periodontitis (S III-S IV)**		*p*
	(*n* = 30, %50)				(*n* = 30, %50)		
	Mean ± SD	Median (Min-Max)			Mean ± SD	Median (Min-Max)	
No. of teeth	27.07 ± 1.23	27.50 (24.00–28.00) ^a^			25.83 ± 2.04	26.00 (20.00–28.00) ^b^	< 0.009*
Age (years)	28.83 ± 9.94	24.00 (22.00–55.00) ^a^			42.90 ± 9.74	43.50 (18.00–64.00) ^b^	< 0.001*
**Gender**							
Male	10(33.33)				12(40.00)		< 0.789**
Female	20(66.67)				18(60.00)		
	**Healthy (H)**		**Stage III Periodontitis (S III)**		**Stage IV Periodontitis (S IV)**		
	(*n* = 30, %50)		(*n* = 13, %21.7)		(*n* = 17, %28.3)		
	Mean ± SD	Median (Min-Max)	Mean ± SD	Median (Min-Max)	Mean ± SD	Median (Min-Max)	
No. of teeth	27.07 ± 1.23	27.50 (24.00–28.00) ^a^	26.15 ± 2.04	26.00 (22.00–28.00) ^ab^	25.59 ± 2.06	26.00 (20.00–28.00) ^b^	**0.018**†
Age (years)	28.83 ± 9.94	24.00 (22.00–55.00) ^a^	43.69 ± 12.87	45.00 (18.00–64.00) ^b^	42.29 ± 6.84	43.00 (31.00–55.00) ^b^	**< 0.001**†
**Gender**							
Male	10(33.33)		4(30.77)		8(47.06)		< 0.569^***^
Female	20(66.67)		9(69.23)		9(52.94)		

*Mann Whitney U test, **Yates’s correction, ***Pearson’s chi-squared test, n (%), †Kruskal Wallis H-test, a,b: Different letters represent significance, Statistical differences are marked in bold

## Clinical periodontal parameters and biochemical findings

Data from full-mouth measurements and sampling sites are listed in Table 2. In the periodontitis group, the PI, BOP (%), GI, PD, CAL, and MOB values in the full-mouth and at GCF sampling sites were higher than in controls (*p* < 0.001). There were no significant differences in full-mouth measurements between the SIII and SIV subgroups. Local periodontal measurements were similar between groups, except for LMOB, which was higher in the SIV than the SIII subgroup (*p* < 0.001).

**Table 2:** Comparison of clinical parameters among the healthy group and the periodontitis group, and periodontitis subgroups

	**Healthy (H)** (*n* = 30, %50)				**Periodontitis (S III-S IV)** (*n* = 30, %50)		*p*
	Mean ± SD	Median (Min-Max)			Mean ± SD	Median (Min-Max)	
PI	0.22 ± 0.16	0.16 (0.01–0.57)			1.65 ± 0.55	1.52 (0.79–2.73)	< 0.001*
BOP (%)	4.34 ± 3.14	4.16 (0.00- 9.45)			55.05 ± 17.42	54.58 (26.28–93.20)	< 0.001*
GI	0.26 ± 0.19	0.21 (0.00- 0.81)			1.85 ± 0.57	1.76 (0.26–2.74)	< 0.001*
PD	1.51 ± 0.16	1.54 (1.20–1.81)			3.36 ± 0.73	3.08 (2.09–5.30)	< 0.001**
CAL	1.52 ± 0.16	1.54 (1.20–1.83)			4.16 ± 0.98	3.99 (3.02–7.68)	< 0.001*
MOB	0.00 ± 0.01	0.00 (0.00- 0.03)			0.38 ± 0.23	0.36 (0.00- 1.21)	< 0.001*
LPI	0.11 ± 0.31	0.00 (0.00- 1.67)			1.76 ± 0.78	1.67 (0.17- 3.00)	< 0.001*
LBOP (%)	5.00 ± 8.91	0.00 (0.00- 33.33)			76.66 ± 19.38	83.33 (33.33–100.00)	< 0.001*
LGI	0.37 ± 0.43	0.17 (0.00- 1.67)			2.13 ± 0.66	2.17 (0.00–3.00)	< 0.001*
LPD	1.73 ± 0.33	1.83 (1.00- 2.33)			5.84 ± 1.22	5.92 (2.83–8.17)	< 0.001*
LCAL	1.74 ± 0.34	1.83 (1.00- 2.33)			6.82 ± 1.37	6.75 (4.17–9.67)	< 0.001*
LMOB	0.01 ± 0.04	0.00 (0.00- 0.20)			0.70 ± 0.66	0.50 (0.00- 2.75)	< 0.001*
	**Healthy (H)** (*n* = 30, %50)		**Stage III Periodontitis (S III)** (*n* = 13, %21.7)		**Stage IV Periodontitis (S IV)** (*n* = 17, %28.3)		*p*
	Mean ± SD	Median (Min-Max)	Mean ± SD	Median (Min-Max)	Mean ± SD	Median (Min-Max)	
PI	0.22 ± 0.16	0.16 (0.01–0.57) ^a^	1.64 ± 0.53	1.56 (0.98–2.53) ^b^	1.67 ± 0.59	1.48 (0.79–2.73) ^b^	**< 0.001**†
BOP (%)	4.34 ± 3.14	4.16 (0.00- 9.45) ^a^	55.28 ± 16.74	55.33 (26.28–76.78) ^b^	54.88 ± 18.43	51.28 (29.71–93.20) ^b^	**< 0.001**†
GI	0.26 ± 0.19	0.21 (0.00- 0.81) ^a^	1.77 ± 0.58	1.74 (0.26–2.61) ^b^	1.91 ± 0.57	2.11 (0.91–2.74) ^b^	**< 0.001**†
PD	1.51 ± 0.16	1.54 (1.20–1.81) ^a^	3.12 ± 0.62	2.83 (2.64–4.79) ^b^	3.54 ± 0.78	3.51 (2.09–5.30) ^b^	**< 0.001**†
CAL	1.52 ± 0.16	1.54 (1.20–1.83) ^a^	3.79 ± 0.62	3.52 (3.13–5.04) ^b^	4.45 ± 1.13	4.35 (3.02–7.68) ^b^	**< 0.001**†
MOB	0.00 ± 0.01	0.00 (0.00- 0.03) ^a^	0.25 ± 0.14	0.25 (0.00- 0.50) ^b^	0.47 ± 0.24	0.40 (0.11–1.21) ^b^	**< 0.001**†
LPI	0.11 ± 0.31	0.00 (0.00- 1.67) ^a^	1.54 ± 0.69	1.50 (0.17–2.67) ^b^	1.93 ± 0.82	2.17 (0.67- 3.00) ^b^	**< 0.001**†
LBOP (%)	5.00 ± 8.91	0.00 (0.00- 33.33) ^a^	78.20 ± 19.70	83.33 (33.33–100.00) ^b^	75.49 ± 19.65	66.66 (50.00- 100.00) ^b^	**< 0.001**†
LGI	0.37 ± 0.43	0.17 (0.00- 1.67) ^a^	1.82 ± 0.67	1.83 (0.00–3.00) ^b^	2.36 ± 0.57	2.67 (1.33- 3.00) ^b^	**< 0.001**†
LPD	1.73 ± 0.33	1.83 (1.00- 2.33) ^a^	5.45 ± 1.01	5.33 (3.67–7.33) ^b^	6.15 ± 1.31	6.33 (2.83–8.17) ^b^	**< 0.001**†
LCAL	1.74 ± 0.34	1.83 (1.00- 2.33) ^a^	6.03 ± 1.21	5.67 (4.17–8.33) ^b^	7.43 ± 1.18	7.67 (5.33–9.67) ^b^	**< 0.001**†
LMOB	0.01 ± 0.04	0.00 (0.00- 0.20) ^a^	0.24 ± 0.21	0.20 (0.00- 0.67) ^b^	1.05 ± 0.67	0.83 (0.20–2.75) ^c^	**< 0.001**†

Abbreviations: PI: plaque index; BOP (%): bleeding on probing; GI: gingival index; PD: probing depth; CAL: clinical attachment loss; MOB; mobility; LPI: localized plaque index; LBOP (%): localized bleeding on probing; LGI: localized gingival index; LPD: localized probing depth; LCAL: localized clinical attachment loss; LMOB: localized mobility

^*^Mann-Whitney U-test, ^**^Independent Samples t-Test, †Kruskal Wallis-H test, a-c: Different letters represent significance. Statistical differences are marked in bold

**Fig. 1:**
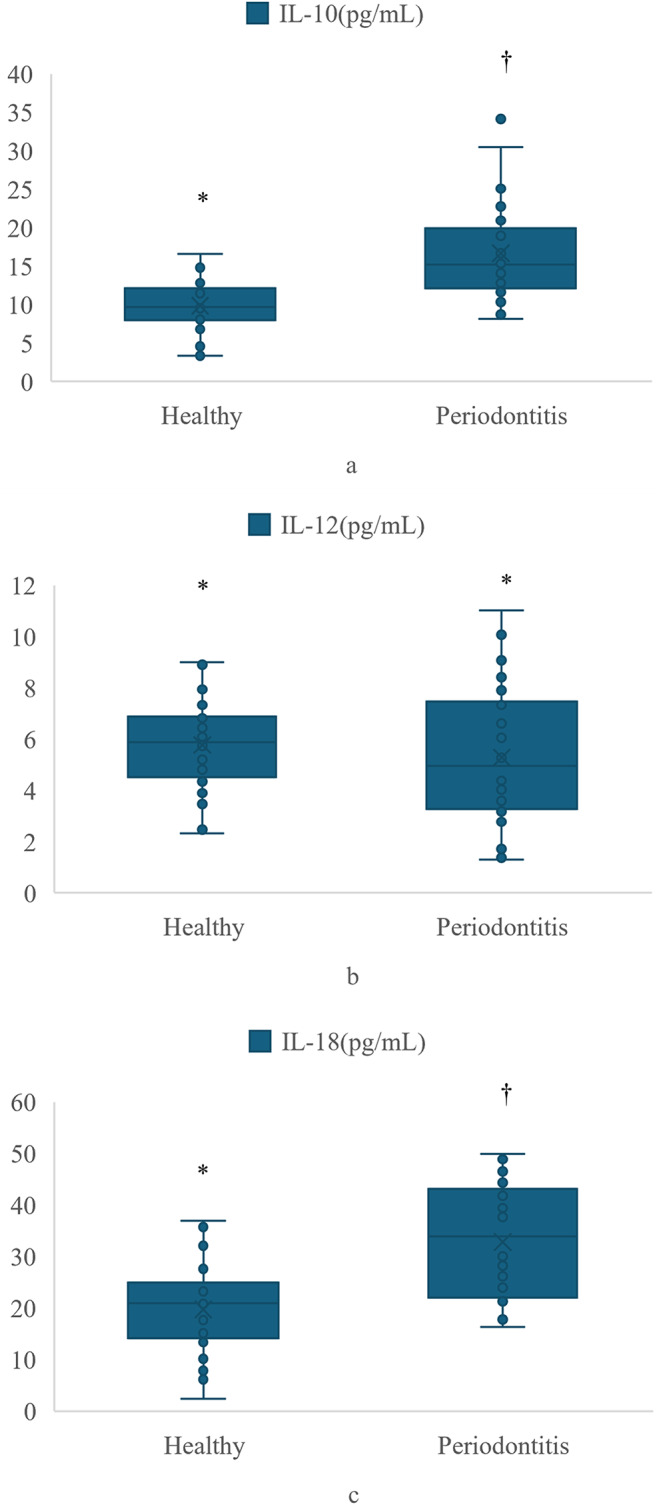
(**a**) Distribution of IL-10 levels in the healthy group and the periodontitis group. (**b**) Distribution of IL-12 levels in the healthy group and the periodontitis group. (**c**) Distribution of IL-18 levels in the healthy group and the periodontitis group Abbreviations: IL, interleukin; pg, picogram; mL, millilitre. *,†: Different symbols represent significance

The biochemical findings are shown in Fig. 1; Table 3. There were no significant differences in IL-12 levels among the groups (*p* = 0.413). The IL-10 and IL-18 levels were significantly higher in the SIII and SIV subgroups than in controls (*p* < 0.001 and *p* < 0.001, respectively), but did not differ significantly between the SIII and SIV subgroups. No differences were found among the groups with respect to the IL-18/IL-10 ratio (*p* = 0.636 and *p* = 0.821, respectively).

**Table 3:** Comparison of biochemical findings among the healthy group and the periodontitis group, and periodontitis subgroups

	**Healthy (H)** (*n* = 30, %50)				**Periodontitis (S III-S IV)** (*n* = 30, %50)		*p*
	Mean ± SD	Median (Min-Max)			Mean ± SD	Median (Min-Max)	
IL-10(pg/mL)	9.85 ± 3.38	9.66 (3.35–16.62)			16.65 ± 6.26	15.20 (8.17–34.14)	**< 0.001**†
IL-12(pg/mL)	5.77 ± 1.70	5.88 (2.31–9.01)			5.29 ± 2.71	4.96 (1.29–11.03)	0.4132‡
IL-18(pg/mL)	19.72 ± 9.19	20.95 (2.43- 37.00)			32.85 ± 11.06	33.91 (16.33–50.01)	**< 0.001**†
IL-18/IL-10	2.34 ± 1.62	1.88 (0.25–8.25)			2.17 ± 0.96	1.98 (0.61–4.62)	0.636
	**Healthy (H)** (*n* = 30, %50)		**Stage III Periodontitis (S III)** (*n* = 13, %21.7)		**Stage IV Periodontitis (S IV)** (*n* = 17, %28.3)		*p*
	Mean ± SD	Median (Min-Max)	Mean ± SD	Median (Min-Max)	Mean ± SD	Median (Min-Max)	
IL-10(pg/mL)	9.85 ± 3.38 ^a^	9.66 (3.35–16.62)	16.80 ± 6.97 ^b^	15.34 (8.70- 34.14)	16.54 ± 5.88 ^b^	15.07 (8.17–30.52)	< 0.001*
IL-12(pg/mL)	5.77 ± 1.70	5.88 (2.31–9.01)	4.55 ± 2.55	3.59 (1.69–11.03)	5.85 ± 2.77	6.12 (1.29–10.07)	0.2952**
IL-18(pg/mL)	19.72 ± 9.19	20.95 (2.43- 37.00) ^a^	34.24 ± 9.53	37.90 (19.10- 48.92) ^b^	31.78 ± 12.28	29.80 (16.33–50.01) ^b^	< 0.001*
IL-18/IL-10	2.34 ± 1.62	1.88 (0.25–8.25)	2.21 ± 0.74	2.12 (1.26–3.44)	2.14 ± 1.12	1.85 (0.61–4.62)	0.821

Abbreviations: IL: interleukin; pg: picogram; mL: millilitre

†Mann-Whitney U-test, ‡Independent Samples t-Test, *Kruskal Wallis-H test, **One-way ANOVA, a-b: Different letters represent significance. Statistical differences are marked in bold

### Correlations

The correlations between clinical periodontal measurements and biochemical findings in the SIII and SIV subgroups, and the periodontitis group are presented in Table 4; Fig. SI-1, and SI-2. In the SIII group, there was no correlation between IL-10 and full-mouth and local clinical measurements (*p* > 0.050). There was a strong positive correlation between IL-12 and LPD and between IL-18 and BOP and PD. The sole significant and strongly negative correlation is observed between the IL-18/IL-10 ratio and IL-10 (*r* = -0.621; *p* = 0.024). In the SIV group, MOB and IL-10 were moderately positively correlated (*r* = 0.505; *p* = 0.039). When examining biomarkers, a significant positive correlation was found between the IL-18/IL-10 ratio and BOP (*r* = 0.672; *p* = 0.003). Furthermore, the IL-18/IL-10 ratio had a moderately negative correlation with IL-10 and a strong positive correlation with IL-18 (*r* = -0.581; *p* = 0.014 and *r* = 0.664; *p* = 0.004, respectively). In the periodontitis group, IL-10 was not correlated with the clinical periodontal parameters, whereas IL-12 was moderately positively correlated with PD, LPD, and LCAL. A moderately positive correlation was found between IL-18 and BOP (*r* = 0.463; *p* = 0.01). The IL-18/IL-10 ratio and BOP exhibited a moderately positive correlation, whereas GI demonstrated a weak but statistically significant positive correlation (*r* = 0.523; *p* = 0.003 and *r* = 0.384; *p* = 0.036, respectively). Upon analysing the IL-18/IL-10 ratio through biomarkers, it was found that there is a moderately negative correlation with IL-10 and a moderately positive correlation with IL-18 (*r* = -0.528; *p* = 0.003 and *r* = 0.595; *p* = 0.001, respectively).

**Table 4:** Correlations between IL-10, IL-12 and IL-18 and clinical periodontal parameters in the periodontitis group, and periodontitis subgroups

**Periodontitis (S III-S IV)**		BOP (%)	GI	PD	LPD	LCAL	IL-10(pg/mL)	IL-12(pg/mL)	IL-18(pg/mL)
IL-10(pg/mL)	r	0.01**	-0.242**	0.099**	-0.016**	0.027**			
	*p*	0.957	0.197	0.604	0.931	0.888			
IL-12(pg/mL)	r	0.042*	0.058*	0.4*	0.548*	0.452*	0.341**		
	*p*	0.826	0.759	**0.029**	**0.002**	**0.012**	0.065		
IL-18(pg/mL)	r	0.463**	0.288**	0.257**	-0.045**	-0.116**	0.311**	-0.115**	
	*p*	**0.01**	0.123	0.17	0.815	0.541	0.094	0.546	
IL-18/IL-10	r	0.523*	0.384*	0.254*	0.02*	-0.134*	-0.528**	0.072*	0.595**
	*p*	**0.003**	**0.036**	0.176	0.916	0.481	**0.003**	0.704	**0.001**
**Stage III Periodontitis (S III)**		BOP (%)	PD	LPD			IL-10(pg/mL)	IL-12(pg/mL)	IL-18(pg/mL)
IL-10(pg/mL)	r	0.484*	0.536**	0.325*					
	*p*	0.094	0.059	0.279					
IL-12(pg/mL)	r	0.139*	0.011**	0.646*			-0.265*		
	*p*	0.652	0.971	**0.017**			0.381		
IL-18(pg/mL)	r	0.747*	0.646**	0.398*			0.49*	0.166*	
	*p*	**0.003**	**0.017**	0.178			0.089	0.589	
IL-18/IL-10	r	0.211*	-0.066**	-0.077*			-0.621*	0.428*	0.320*
	*p*	0.49	0.83	0.803			**0.024**	0.144	0.287
**Stage IV Periodontitis (S IV)**		MOB					IL-10(pg/mL)	IL-12(pg/mL)	IL-18(pg/mL)
IL-10(pg/mL)	r	0.505**							
	*p*	**0.039**							
IL-12(pg/mL)	r	-0.185**					0.054*		
	*p*	0.476					0.836		
IL-18(pg/mL)	r	0.281**					0.301**	-0.243**	
	*p*	0.274					0.24	0.348	
IL-18/IL-10	r	0.672*					-0.581*	-0.071*	0.664**
	*p*	**0.003**					**0.014**	0.787	**0.004**

Abbreviations: IL: interleukin; BOP (%): bleeding on probing; PD: probing depth; MOB; mobility; LPD: localized probing depth; LCAL: localized clinical attachment loss; pg: picogram; mL: millilitre

*Pearson correlation coefficient, **Spearman’s rho correlation coefficient. Statistical differences are marked in bold

### Logistic regression

The associations among the measurements calculated using a univariate model of binary logistic regression are listed in Table 5. Age and GI, MOB, LPI, LGI, and LMOB were associated with periodontitis group. LMOB was strongly associated with periodontitis group. IL-12 and IL-18/IL-10 ratio was not correlated with periodontitis group. There were significant associations between the periodontitis group and IL-10 and IL-18 levels.

**Table 5:** Binary logistic regression

	Healthy (H) (*n* = 30, %50)	Periodontitis (S III-S IV) (*n* = 30, %50)	Univariate	
			OR (%95 CI)	*p*
Gender				
Male	10 (45.5)	12 (54.5)	Reference	
Female	20 (52.6)	18 (47.4)	0.75 (0.262–2.151)	0.592
Age	28.83 ± 9.94	42.90 ± 9.74	1.137 (1.068–1.21)	**< 0.001**
PI	0.22 ± 0.16	1.65 ± 0.55	----	----
BOP (%)	4.34 ± 3.14	55.05 ± 17.42	----	----
GI	0.26 ± 0.19	1.85 ± 0.57	1.950 (1.279–2.973)	**0.002**
PD	1.51 ± 0.16	3.36 ± 0.73	----	----
CAL	1.52 ± 0.16	4.16 ± 0.98	----	----
MOB	0.00 ± 0.01	0.38 ± 0.23	2.043 (1.043–4.002)	**0.037**
LPI	0.11 ± 0.31	1.76 ± 0.78	1.603 (1.273–2.019)	**< 0.001**
LBOP (%)	5.00 ± 8.91	76.66 ± 19.38	----	----
LGI	0.37 ± 0.43	2.13 ± 0.66	1.482 (1.213–1.812)	**< 0.001**
LPD	1.73 ± 0.33	5.84 ± 1.22	----	----
LCAL	1.74 ± 0.34	6.82 ± 1.37	----	----
LMOB	0.01 ± 0.04	0.70 ± 0.66	6.412 (1.998–20.578)	**0.002**
IL-10(pg/mL)	9.85 ± 3.38	16.65 ± 6.26	1.461 (1.19–1.795)	**< 0.001**
IL-12(pg/mL)	5.77 ± 1.70	5.29 ± 2.71	0.907 (0.721–1.141)	0.406
IL-18(pg/mL)	19.72 ± 9.19	32.85 ± 11.06	1.13 (1.059–1.207)	**< 0.001**
IL-18/IL-10	2.34 ± 1.62	2.17 ± 0.96	0.908 (0.614–1.344)	0.630

Abbreviations: S III: Stage III periodontitis; S IV: Stage IV periodontitis; IL: interleukin; PI: plaque index; BOP (%): bleeding on probing; GI: gingival index; PD: probing depth; CAL: clinical attachment loss; MOB; mobility; LPI: localized plaque index; LBOP (%): localized bleeding on probing; LGI: localized gingival index; LPD: localized probing depth; LCAL: localized clinical attachment loss; LMOB; localized mobility; pg: picogram; mL: millilitre; n (%); mean ± S. Deviation; OR: Odds Ratio; CI: Confidence Interval

Statistical differences are marked in bold

## Discussion

Majority of the studies examining the GCF biomarkers were designed according to 1999 classification, to the best of our knowledge, this is the first study to evaluate clinical measurements, GCF IL-10/12/18 levels, IL-18/IL-10 ratio and their correlations within the same population in healthy controls and subjects with periodontitis using the 2017 classification system. The results of the study indicated a positive correlation between IL-18 pro-inflammatory activity and pocket depth. Conversely, IL-10 exhibited anti-inflammatory activity. Moreover, the ratio of the pro-inflammatory cytokine IL-18 to the anti-inflammatory cytokine IL-10 did not show a significant difference between healthy subjects and those with periodontitis.

Periodontal inflammation is associated with changes in the levels of inflammatory inhibitors, which may contribute to the development of severe progressive periodontitis [34]. In this study, the periodontitis group had significantly higher IL-10 levels than the healthy control group. In Gamonal et al., IL-10 was present in 43% of GCF samples from patients with periodontitis [11]. Regarding IL-10 production in pockets with varying probing depths, the authors stated that the correlation between these results and the disease progression is unknown. By contrast, we did not identify any variation in IL-10 level according to periodontitis stage. Consistently, Bostanci et al. reported that individuals with chronic periodontitis (CP) have a higher level of GCF IL-10 than healthy controls before and after periodontal therapy [35]. The authors suggested that this increase in IL-10 may counteract inflammation in patients with CP. In another study, the IL-10 level was higher in moderate and deep pockets than in shallow and control sites and was unaffected by periodontal therapy [36]. According to Lappin et al., Th2 cells produce IL-10 to inhibit IFN-γ production, promoting and maintaining a humoral immune response [37]. Consequently, the presence of IL-10 in the GCF of patients with periodontal disease may modulate the local immune response and have an anti-inflammatory effect. Regarding the other variables, only the SIV subgroup showed a moderate correlation between the IL-10 level and the MOB value. The association between the IL-10 level and tooth mobility in the SIV subgroup might suggest that IL-10 influences the healing of the periodontium. According to our findings and prior reports, IL-10 is related to advanced periodontitis; however, it might not have a destructive effect [11, 27, 35–37].

The pro-inflammatory and immunoregulatory effects of IL-12 promote Th1 immunity and inhibit Th2 immunity and are implicated in the initiation and progression of gingival inflammation [27]. We found no association between IL-12 and other variables in controls and the periodontitis groups. Johnson and Serio hypothesised that IFN-γ secretion is influenced by decreased IL-12 and increased IL-18 concentrations in diseased gingiva [27]. They speculated that there was an inverse correlation between IL-12 concentration and sulcular depth in biopsies and suggested that this contributed to the defective Th1-Th2 shift in periodontal inflammation. To determine the severity of inflammation, Tsai et al. conducted site-specific measurements; the level of IL-12 was greater at periodontitis sites compared to gingivitis and healthy sites [22]. The concentrations of IL-12 differed only minimally among the three sites, which they hypothesised could be attributed to the pathogenesis of periodontitis. In another study, a low level of IL-12 was found in GCF from periodontitis and gingivitis sites, and decreased as inflammation progressed [21]. Yücel et al. reported a higher level of IL-12 in a CP group than in healthy controls [38]. However, the CP group had a greater volume of GCF compared to the gingivitis and control groups, and the IL-12 concentrations did not significantly differ among the groups. IL-12 stimulates mesenchymal stem cells in dental tissues such as gingiva and periodontal ligaments, having an immunoregulatory effect. It induces the production of immunomodulatory proteins, including indoleamine-pyrrole 2,3-dioxygenase and human leukocyte antigen molecules, via an IFN-γ-dependent pathway [24]. The immunomodulatory effect of IL-12 has been confirmed using human periodontal ligament cells from a healthy periodontium [39]. Further research is needed to determine whether its effect is anti-inflammatory or pro-inflammatory. It has been observed that the quantity of GCF is greater in inflamed regions compared to healthy areas [11, 19, 22, 38, 40]. Additionally, several studies have suggested that the findings regarding the inflammatory status of cytokines in this study were consistent, regardless of the total amount and concentration analysed [11, 19, 22, 38].

IL-18 has a proinflammatory effect by increasing the synthesis of nitric oxide, chemokines, and cell adhesion molecules [26]. Its concentration was higher in the periodontitis group than in healthy controls. However, the disparity between the SIII and SIV subgroups was negligible. As periodontal disease advances, there is a proportional increase in the concentration of IL-18 in GCF [25, 33, 41, 42]. In a previous study, periodontal inflammation did not resolve, possible due to the accumulation of IL-18 in diseased gingiva [27]. The authors hypothesised that IL-18 might be a target for preventive and palliative periodontitis treatments, based on a robust correlation between pocket depth and IL-18 concentration. The correlation between IL-18 levels and BOP and PD values in our study is consistent with prior reports [25, 27]. Furthermore, Orozco et al. showed that the IL-18 level in GCF increased with inflammation. In that study, the IL-12 levels at gingivitis and periodontitis sites were low; IL-18 was the predominant cytokine [21]. Although synergism between IL-18 and IL-12 and their mechanism with IFN-γ has been reported, we found no correlation between these cytokines [21–23, 26]. Our findings suggest that IL-18 is a pro-inflammatory cytokine that is significantly associated with periodontitis, and its influence increases as the severity of inflammation increases.

To date, no studies have examined the relationship between the IL-18/IL-10 ratio and periodontal diseases. Given that the anti-inflammatory cytokine IL-10 positively impacts periodontal diseases and the pro-inflammatory cytokine IL-18 is associated with the progression of periodontal disease, the concept that the balance of pro-inflammatory and anti-inflammatory cytokines may be a crucial determinant of clinical outcomes has been explored through studying the IL-18/IL-10 ratio [11, 21–23, 25–27, 35–37]. Previous research on acute coronary syndrome concluded that the IL-18/IL-10 ratio had a positive predictive value for disease effects and adverse hospital outcomes [43–45]. In our study’s analyses of both the main group and subgroups, the IL-18/IL-10 ratio did not emerge as a significant predictor of periodontitis risk, despite exhibiting a low-level influence on certain clinical values. Nonetheless, this finding may prompt further exploration, suggesting additional research is required.

Prior studies have used a variety of case definitions for periodontal diseases. The 2017 Classification of Periodontal and Peri-implant Diseases and Conditions points out the importance of establishing a definitive correlation between cytokines and periodontal diseases [6, 46]. In our study, we selected SIII-SIV periodontitis to compare the outcomes of subjects with chronic and aggressive periodontitis, given that SI-SII periodontitis represents the boundary between gingivitis and periodontitis, and attachment loss and destruction are minimal. There may be differences in treatment complexity and response between these stages but our results revealed no differences in biochemical parameters. As per the 2017 classification, the primary criteria for grade determination is the loss in 5-year radiographic measurements and the potential of biomarkers to function as grade modifiers is also emphasized in the new classification [6]. However, longitudinal studies with extended follow-up periods are needed to assess direct correlations between grade and biomarkers. Additionally, the age restriction of this study may have precluded identification of SIV subjects who met the GB criteria.

In addition to achieving the primary outcomes regarding the IL values between the healthy and periodontitis groups, our study has provided us with the opportunity to conduct an evaluation between various stages of periodontitis (Stage III and Stage IV). This assessment has furnished additional insights that may be implemented in future studies.

This study had several limitations. First, it was cross-sectional, precluding assessment of the post-treatment state. Second, our results are not generalisable to all patients with periodontitis because the subjects were non-smokers and systemically healthy. A notable aspect of this study was the comprehensive evaluation of multiple biomarkers and the division of the subjects into groups defined according to a recent classification.

## Conclusion

Elevated IL-18 levels in subjects with periodontitis suggest a role in unresolved periodontal inflammation. The strong correlation between IL-18 and PD makes it a potential target for preventive and therapeutic interventions in periodontitis. Conversely, IL-12 exhibited complex associations, necessitating further investigation of its function in the progression of periodontitis. Our results will pave the way for future longitudinal investigations involving a more diverse population to provide insight into cytokine dynamics in periodontal diseases.

### Electronic supplementary material

Below is the link to the electronic supplementary material.

Supplementary Material 1

## Data Availability

The data supporting the findings of this study are available from the corresponding author upon reasonable request.
