# Supplementary material for: Associations between interleukin-10, -12, and − 18 and periodontal health and disease: a cross-sectional study
Source: Clin Oral Investig. 2024 Jul 31;28(8):458. doi: 10.1007/s00784-024-05843-8 (PMC11289060; doi:10.1007/s00784-024-05843-8)
Supplement: Supplementary file 1 — Supplementary Material 1 [file 784_2024_5843_MOESM1_ESM.docx]

**Associations Between Interleukin-10, -12, and -18 and Periodontal Health and Disease: A Cross-Sectional Study**

**Clinical Oral Investigation Journal**

Elif Ilke CEBESOY ^1,2^ DDS, PhD (EIC) e-mail: dtelifilke@hotmail.com

Müge ALTACA ^1,2^ DDS, PhD (MA) e-mail: mugeergunn@gmail.com

Necla Asli KOCAK-OZTUG ^1,3^ DDS, PhD (NAK-O) e-mail: asli.kocak@istanbul.edu.tr

Ilknur BINGÜL ^4^ PhD, Assoc. Prof. (IB) e-mail: ilknur.bingul@istanbul.edu.tr

Emine CIFCIBASI ^1^ DDS, PhD, Assoc. Prof. (EC) e-mail: emineyek@istanbul.edu.tr

^1^ Faculty of Dentistry, Department of Periodontology, Istanbul University, Istanbul 34116, Turkey.

^2^ Institute of Graduate Studies in Health Sciences, Department of Periodontology, Istanbul University, Istanbul 34126, Turkey.

^3^ School of Dentistry, Faculty of Health and Behavioural Sciences, The University of Queensland, Brisbane, Queensland 4006, Australia

^4^ Department of Medical Biochemistry, Istanbul Faculty of Medicine, Istanbul University, Istanbul, Turkey

**Corresponding author:**

Dr. Emine Cifcibasi

Department of Periodontology

Faculty of Dentistry

Istanbul University

34116 Istanbul, Turkey

Tel: +90 530 784 8581

Fax: +90 212 531 2230

E-mail: [emineyek@istanbul.edu.tr](mailto:emineyek@istanbul.edu.tr)

Supporting Information.1 a. The correlations between clinical periodontal measurements and the biochemical findings in the S III group. b. The correlations between clinical periodontal measurements and the biochemical findings in the S IV group. c. The correlations between clinical periodontal measurements and the biochemical findings in the periodontitis group

Abbreviations: S III, Stage III periodontitis; S IV, Stage IV periodontitis; IL, interleukin; PI, plaque index; BOP (%): bleeding on probing; GI, gingival index; PD, probing depth; CAL, clinical attachment loss; MOB; mobility; LPI, localized plaque index; LBOP (%): localized bleeding on probing; LGI, localized gingival index; LPD, localized probing depth; LCAL, localized clinical attachment loss; LMOB; localized mobility; pg, picogram; mL, millilitre.

Green colour represents positive correlation. Red colour represents negative correlation.


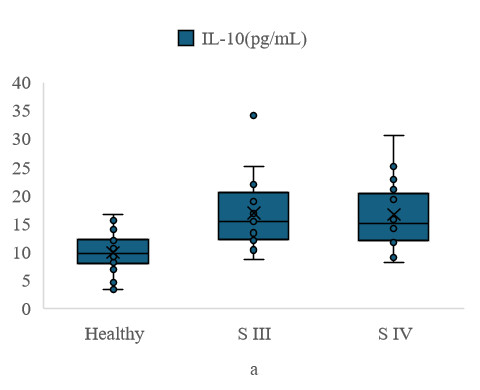

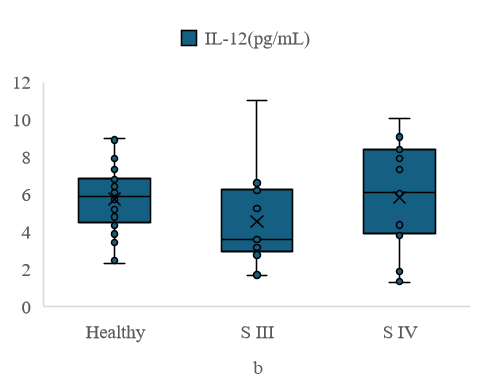

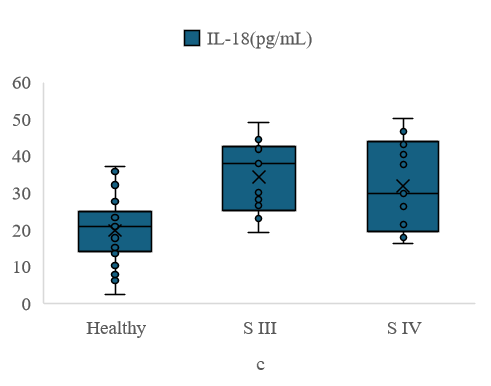


*

*

*

*

*

†

†

†

†

Supporting Information.2 a. Distribution of IL-10 levels in the H group and S III and S IV periodontitis groups. b. Distribution of IL-12 levels in the H group and S III and S IV periodontitis groups. c. Distribution of IL-18 levels in the H group and S III and S IV periodontitis groups.

Abbreviations: S III, Stage III periodontitis; S IV, Stage IV periodontitis; IL, interleukin; pg, picogram; mL, millilitre.

*,†: Different symbols represent significance.
